# Sustained Release of Insulin-Like Growth Factor-1 from *Bombyx mori* L. Silk Fibroin Delivery for Diabetic Wound Therapy

**DOI:** 10.3390/ijms22126267

**Published:** 2021-06-10

**Authors:** Meng-Jin Lin, Mei-Chun Lu, Hwan-You Chang

**Affiliations:** 1 Miaoli District Agricultural Research and Extension Station, Council of Agriculture, Executive Yuan, Miaoli 363201, Taiwan; Lmj@mdais.gov.tw (M.-J.L.); Lumj@mdais.gov.tw (M.-C.L.); 2 Institute of Molecular Medicine, National Tsing Hua University, Hsinchu 300044, Taiwan

**Keywords:** *Bombyx mori* L., insulin-like growth factor-1 (IGF-1), silk fibroin, wound healing, diabetes

## Abstract

The goals of this study are to develop a high purity patented silk fibroin (SF) film and test its suitability to be used as a slow-release delivery for insulin-like growth factor-1 (IGF-1). The release rate of the SF film delivering IGF-1 followed zero-order kinetics as determined via the Ritger and Peppas equation. The release rate constant was identified as 0.11, 0.23, and 0.09% h^−1^ at 37 °C for SF films loaded with 0.65, 6.5, and 65 pmol IGF-1, respectively. More importantly, the IGF-1 activity was preserved for more than 30 days when complexed with the SF film. We show that the IGF-1-loaded SF films significantly accelerated wound healing in vitro (BALB/3T3) and in vivo (diabetic mice), compared with wounds treated with free IGF-1 and an IGF-1-loaded hydrocolloid dressing. This was evidenced by a six-fold increase in the granulation tissue area in the IGF-1-loaded SF film treatment group compared to that of the PBS control group. Western blotting analysis also demonstrated that IGF-1 receptor (IGF1R) phosphorylation in diabetic wounds increased more significantly in the IGF-1-loaded SF films group than in other experimental groups. Our results suggest that IGF-1 sustained release from SF films promotes wound healing through continuously activating the IGF1R pathway, leading to the enhancement of both wound re-epithelialization and granulation tissue formation in diabetic mice. Collectively, these data indicate that SF films have considerable potential to be used as a wound dressing material for long-term IGF-1 delivery for diabetic wound therapy.

## 1. Introduction

The skin is a large organ that plays an essential role in maintaining homeostasis and also protecting the body from microorganism invasion. Severe skin damage can result in infections, loss of body temperature and fluid balance, and often death [1,2]. Chronic wounds accompanied by inflammation and delayed wound repair commonly cause the disruption of the period of wound healing [3], which consumes great resources of healthcare. More than 6.5 million chronic skin ulcer cases primarily caused by blood pressure, venous stasis, or diabetes mellitus are reported annually in the United States, with treatment costs estimated to exceed USD 20 billion [4,5]. Although a number of products have been developed to improve chronic wound healing, no efficient therapy has been applied so far [6].

Growth factors have been extensively studied for their efficacy in accelerating chronic wound repair [7,8], with the majority of research focusing on epidermal growth factor, fibroblast growth factors, platelet-derived growth factor, vascular endothelial growth factor, and insulin-like growth factors. Many studies have revealed the potential of these growth factors to improve wound closure, granulation tissue formation, and angiogenesis [9]. A gel formulation of recombinant platelet-derived growth factor-BB (REGRANEX^®^) was approved for treating diabetic ulcers in the United States and Europe [10]. Despite their effectiveness, many growth factors exhibit a relatively short half-life in vivo, presumably due to enzymatic degradation in the wound bed [11,12]. To maintain an effective concentration of growth factors, a formulation of the factors combined with a suitable delivery vehicle, such as a liposome, micelle, dendrimer, or protein-based drug delivery system, is suggested [13,14,15].

Insulin-like growth factor-1 (IGF-1) is a 7.6 kDa polypeptide with three intramolecular disulfide bridges. IGF-1 has been approved by the FDA for the treatment of Growth Hormone Insensitivity Syndromes [16]. In diabetic wounds, low expression of IGF-1 in the basal layer and fibroblasts has been proposed to contribute to delayed healing [17]. In addition, previous reports demonstrated that IGF-1 signaling improved the epithelialization and vascularization of wounds [18,19]. Nevertheless, because of the short half-life (<15 min), IGF-1 is usually given as a continuous infusion that limits its clinical usefulness [20].

Silk fibroin (SF) from the *Bombyx mori* L. silkworm is an FDA-approved polymer characterized by its biocompatibility, biodegradability, and low immunogenicity [21,22]. Different forms of SF, including films, tubes, gels, particles, microneedles, and electrospun nanofibers, have been used in the controlled release of growth factors [23,24,25]. Reportedly, SF crystallinity and structure are crucial for the better control of the linearity of the growth factor releasing rate [24]. The diffusion rate of the drugs in SF with a structure of porous hydrogel networks is distinct from that of hydrophobic fibroin films [26,27]. Aside from matrix porosity and hydrophobicity, the nature of cargo molecules, including their charge, shape, and size, also affects a drug’s releasing rate [28]. Thus, the actual release rates of different growth factors loaded into SF matrices need to be analyzed individually.

**Figure 1:**
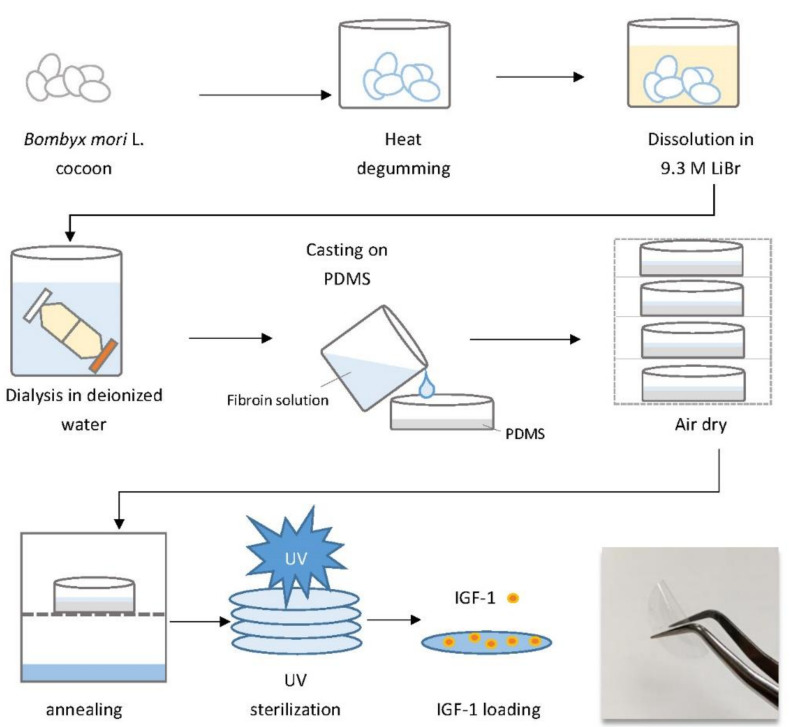
Schematic representation of the stepwise procedure of insulin-like growth factor 1 (IGF-1)-loaded silk fibroin film (IGF-1-loaded SF film) fabrication.

The slow release of IGF-1 from a freeze-dried porous SF scaffold has been demonstrated in vitro. The total cumulative IGF-I release after 29 days was approximately 26% of the initial loading, and the release model did not follow the zero-order kinetics [29]. Additionally, the beneficial effect of the slow-release effect in tissue engineering and muscle therapy applications has also been demonstrated in previous studies [30,31,32]. Nevertheless, the release kinetics of IGF-1 loaded at different dosages into an SF film, as well as the in vivo biological potency of released IGF-1 on chronic diabetic wound healing, are unknown. Therefore, the aim of this study was to determine the release kinetics and dosage effect of IGF-1 loaded onto an SF film, which was performed with a novel patent pending procedure (Figure 1), as well as to evaluate the healing effectiveness and gene expression of a diabetic wound treated with either an IGF-1-loaded SF film or an IGF-1-loaded commercial dressing. The results of the current study can assist in developing an appropriate growth factor delivery vehicle, as well as a chronic wound dressing, both of which are currently lacking in clinical application.

## 2. Results

### 2.1. In Vitro Release Studies

**Figure 2:**
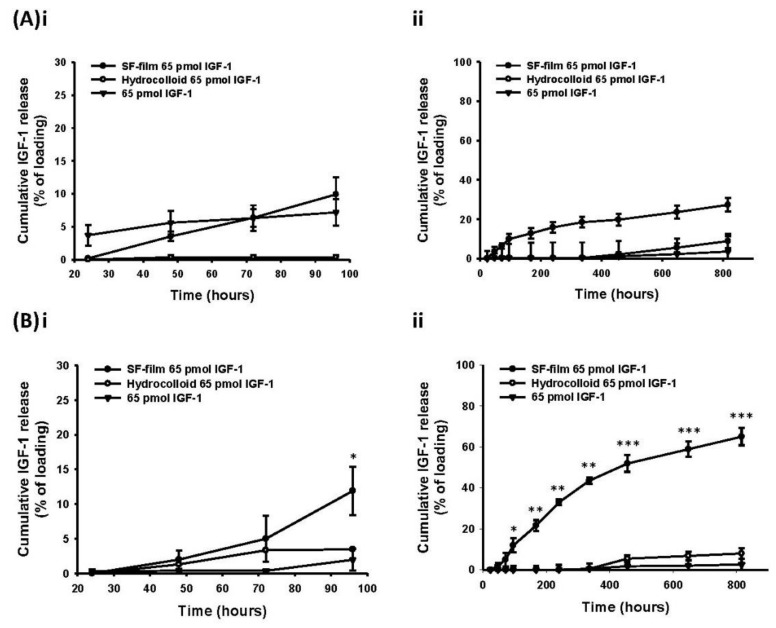
Release profiles of insulin-like growth factor 1 (IGF-1)-loaded silk fibroin film (IGF-1-loaded SF film), IGF-1 loaded onto SF films or hydrocolloid dressing. (**A**) The cumulative release curve of 65 pmol IGF-1 (i) from 24 to 96 h, and (ii) 24 to 816 h at 4 °C. (**B**) The cumulative release curve of 65 pmol IGF-1 (i) from 24 to 96 h, and (ii) 24 to 816 h at 37 °C. (●) SF films loaded with IGF-1; (◯) Hydrocolloid loaded with IGF-1; (▼) IGF-1 alone, * *p* < 0.05, ** *p* < 0.01, *** *p* < 0.001 in comparison with IGF-1 alone treatments (*n* = 3, ± SEM) using a Dunnett’s *t* test.

Hydrocolloid, owing to its high wound drainage absorption and moisture maintaining abilities, is a common dressing material for wound repair [33]. The study used hydrocolloid as a control to investigate the effectiveness of SF films on drug release and wound healing. We determined the IGF-1 release rate from SF films or hydrocolloid dressings over a period of more than 30 days. SF films were first impregnated with 0.65, 6.5, or 65 pmol IGF-1 in a final volume of 5 mL. In all cases, all of the IGF-1 was adsorbed onto the matrix as determined by measuring residual IGF-1 in the solution using an ELISA kit. The IGF-1 loaded films were then transferred into 5 mL of PBS to determine the release rate of IGF-1. The hydrocolloid dressing exhibited a strong IGF-1 binding activity; less than 10% of the bound IGF-1 was released after incubation in PBS for 34 days either at 4 °C or 37 °C. Cumulative IGF-1 release from SF films loaded with 0.65, 6.5, or 65 pmol samples was found to be approximately 14%, 16%, and 28%, respectively, after 34 days at 4 °C (Figure 2A, Figure S1A,C). The IGF-1 release rate at 37 °C was significantly faster than that at 4 °C, showing a cumulative release of 93.2%, 88.9%, and 65.1% for SF films loaded with 0.65, 6.5, or 65 pmol IGF-1, respectively, at day 34 (Figure 2B, Figure S1B,D). Free 65 pmol IGF-1 was only detected within 24 h, reflecting its short half-life.

The kinetic release profiles of SF films illustrated a zero-order release mechanism either in the storage (4 °C) or in reaction environments (37 °C). The SF films loaded with 0.65 pmol IGF-1 displayed a release constant (K) of 0.03% h^−1^, release exponent (*n*) of 1.03, and regression coefficient (R^2^) value of 0.92 between 48 and 816 h at 4 °C. The values of the same parameters (K, *n* and R^2^) for the same time period for SF films loaded with 6.5 pmol IGF-1 were (K = 0.05% h^−1^; *n* = 0.97, and R^2^ = 0.96) and with 65 pmol (K = 0.06% h^−1^; *n* = 1.02, R^2^ = 0.88). The results also demonstrated that SF films loaded with 0.65 or 6.5 pmol IGF-1 were almost completely released by the end of 816 h.

At 37 °C, SF films loaded with 0.65 pmol (K = 0.11% h^−1^; *n* = 1.21) and 6.5 pmol (K = 0.23% h^−1^; *n* = 1.00) IGF-1 showed zero-order release mechanisms, with regression coefficient (R^2^) values of 0.95 and 0.96, respectively, between 48 and 456 h. A zero-order release mechanism was also found in SF films loaded with 65 pmol IGF-1 (K = 0.09% h^−1^; *n* = 1.05; R^2^ = 0.98) between 48 and 816 h at 37 °C (Table 1). This study noted that the complete release of 6.5 pmol adsorbed on the SF film could be achieved during the 30-day period. Higher amounts of IGF-1 (65 pmol) loaded into SF films also maintained rapid exponential release between 48 and 816 h at 37 °C, although this required a longer release time (*t* 50 = 648 h for 50% drug release) for complete release than the films loaded with a lower IGF-1 quantity. These results show that the complete drug release precisely for wound healing was correlated with the amount of the payload in SF film.

### 2.2. In Vitro and In Vivo Wound Healing Studies

**Figure 3:**
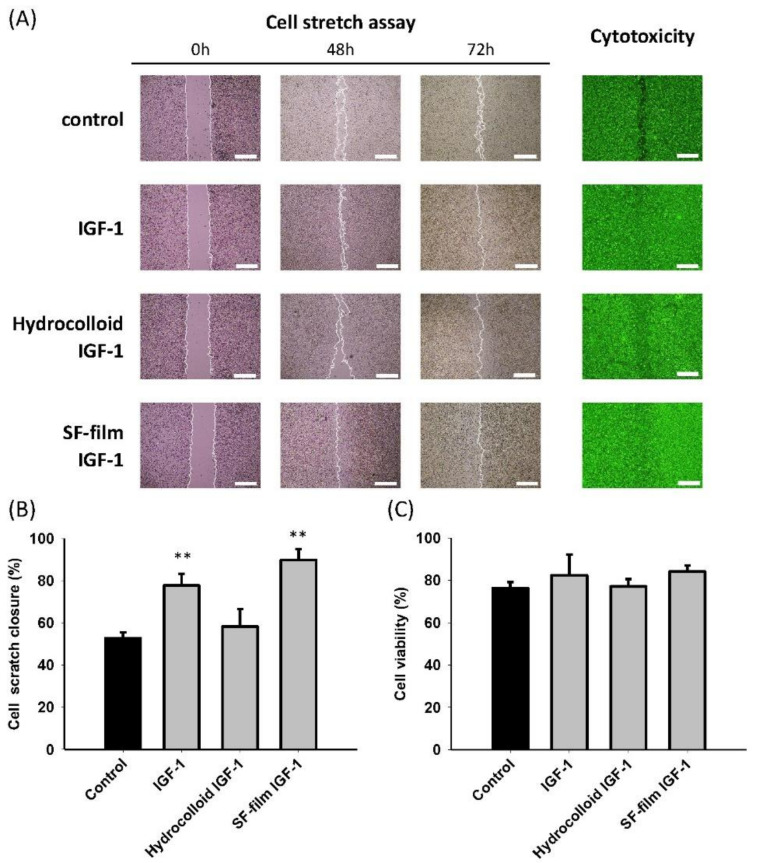
Effects of IGF-1, hydrocolloid IGF-1, and SF film IGF-1 on BALB/3T3 fibroblast scratch closure, cytotoxicity, and viability. (**A**) Scratch closure and cytotoxicity of BALB/3T3 monolayer scratch closure in the presence of hyperglycemic medium at different times. Cells were stained with LIVE/DEAD stain and examined under a fluorescence microscope. (**B**) Quantification of cell scratch closure after different treatment at 48 h in hyperglycemic medium. (**C**) Cell viability in the presence after different treatment at 72 h. Significant differences between the control (black bar) and treatment groups were determined by Dunnett’s multiple comparison post hoc test. ** *p* < 0.01; *n* = 3; mean ± SEM. (Scale bars = 500 μm).

The effects of IGF-1 alone, hydrocolloid IGF-1, and SF film–IGF-1 on scratch closure and viability of BALB/3T3 fibroblasts in hyperglycemic media were evaluated. The scratch closure assay was accelerated by approximately 25% and 37% in the presence of 65 pmol IGF-1 alone or SF film IGF-1 treated for 48 h, respectively (Figure 3A,B). However, the cell cytotoxicity and viability showed no difference with respect to IGF-1, hydrocolloid IGF-1, or SF film IGF-1 treatments (Figure 3A,C).

**Figure 4:**
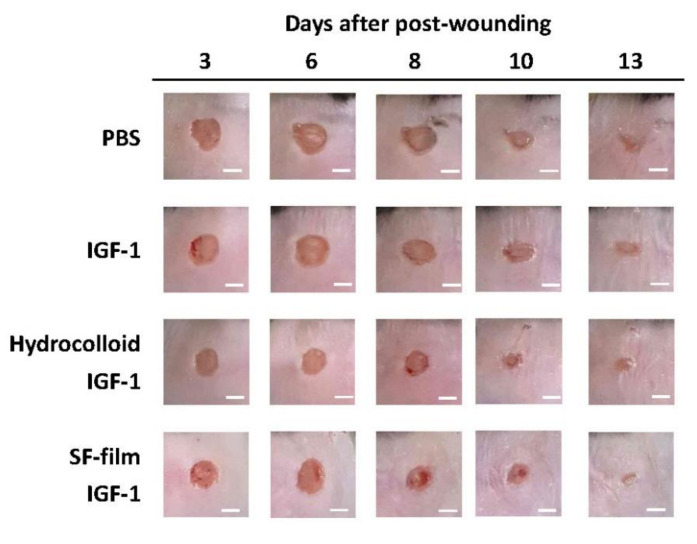
Time course analysis of diabetic wound healing in PBS (control), native insulin-like growth factor 1 (IGF-1), a commercial dressing product (hydrocolloid IGF-1), and insulin-like growth factor 1-loaded silk fibroin film (SF film IGF-1) treatments. Images of the wound area from days 0 to 13 post-wounding (Scale bars = 5 mm).

Using a diabetic mouse as a wound healing model, we further compared the effect of IGF-1-loaded SF films and the hydrocolloid dressing on diabetic wound healing. In this study, we used 8-week-old female leptin-receptor deficient *db*/*db* mice, characterized by hyperglycemia and impaired wound healing, as a model for type II diabetes mellitus. The wound area in all treatment conditions was similar, with more than 50% showing recovery late in the post-wounding period, i.e., after 10 to 13 days of wound treatment. However, compared to the mice which received the PBS treatment (negative control), the external wound area was smaller in the IGF-1-loaded SF film treatment mice (Figure 4).

**Figure 5:**
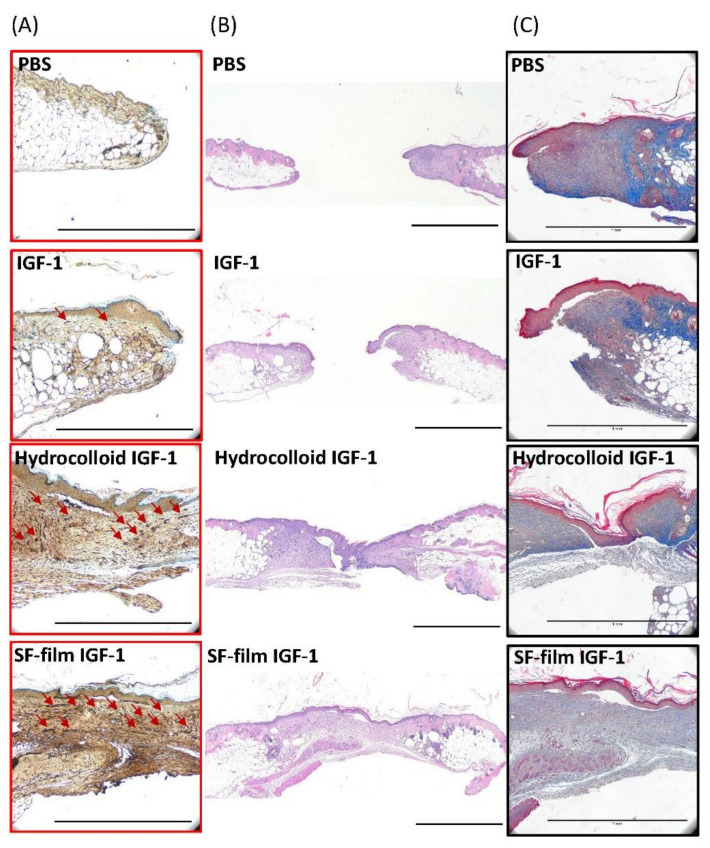
Sections of regenerative tissue in the wound area following treatment with PBS (control), native insulin-like growth factor 1 (IGF-1), a commercial dressing product (hydrocolloid IGF-1), and insulin-like growth factor 1-loaded silk fibroin films (SF film IGF-1) 13 days post-wounding. (**A**) Immunohistological sections of regenerative tissue were stained with an anti-human CD31 antibody. The presence of blood vessels is indicated by red arrows, (**B**) Hematoxylin and Eosin (H&E) stain, and (**C**) Masson’s trichrome stain (black frame). (Scale bars = 1 mm).

To determine the degree of wound closure in diabetic mice, we removed the wound tissue after 13 days of treatment and examined sections of the wound tissue using H&E staining. Extracellular matrix composition in the wound bed was further evaluated using Masson’s trichrome staining, and vessel density was confirmed using a CD31 immunohistochemistry stain. As shown in Figure 5A–C, wound closure was faster with the application of hydrocolloid IGF-1 or SF film IGF-1 treatments, compared to PBS or IGF-1 treatments alone. Compared to hydrocolloid IGF-1 treatment, SF film IGF-1-treated wounds displayed relatively smooth surfaces, suggesting an enhancement of angiogenesis in the diabetic wounds.

**Figure 6:**
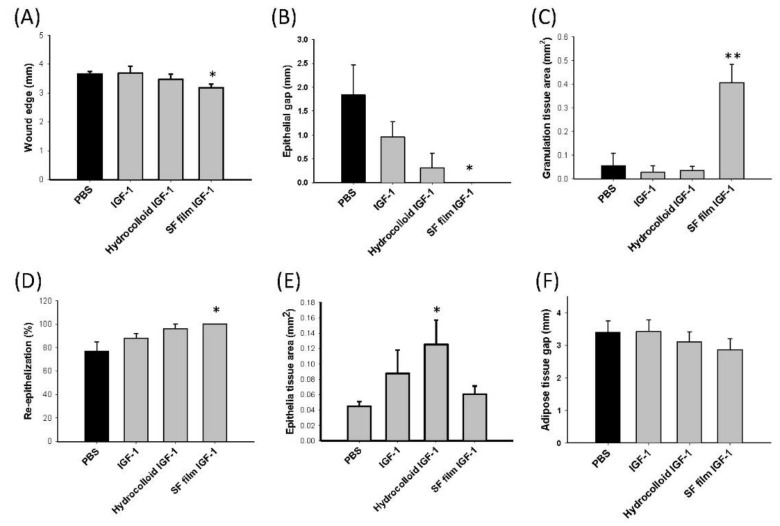
Analysis of tissue regeneration in diabetic wounds treated with PBS (control), native insulin-like growth factor 1 (IGF-1), a commercial dressing product (hydrocolloid IGF-1), and insulin-like growth factor 1-loaded silk fibroin films (SF film IGF-1). (**A**) Quantification of wound edge distance, (**B**) epithelial gap, (**C**) granulation tissue area, (**D**) re-epithelialization, (**E**) epithelial tissue area, and (**F**) adipose tissue gap following wound healing 13 days post-wounding. Significant differences between PBS (black bar) and other treatments were determined using Dunnett’s multiple comparison post hoc test. * *p* < 0.05, ** *p* < 0.01; *n* = 5; ±SEM.

As results have previously shown, the wound edge and epithelial gap in *db*/*db* mice administered with IGF-1-loaded SF films were improved by 13.2% and 23.0%, respectively, both of which were smaller than PBS control mice for the same skin repair period (Figure 6A,B). Furthermore, IGF-1-loaded SF films displayed a significantly higher cell proliferation activity, by approximately six times higher than the PBS control group in the granulation tissue area (Figure 6C). The highest re-epithelization was shown in SF film IGF-1 treatments (Figure 6D), while the epithelial tissue in the hydrocolloid IGF-1 group was clearly thicker than the PBS control group (Figure 6E). For all treatments, no significant differences were observed in the adipose tissue gap (Figure 6F).

**Figure 7:**
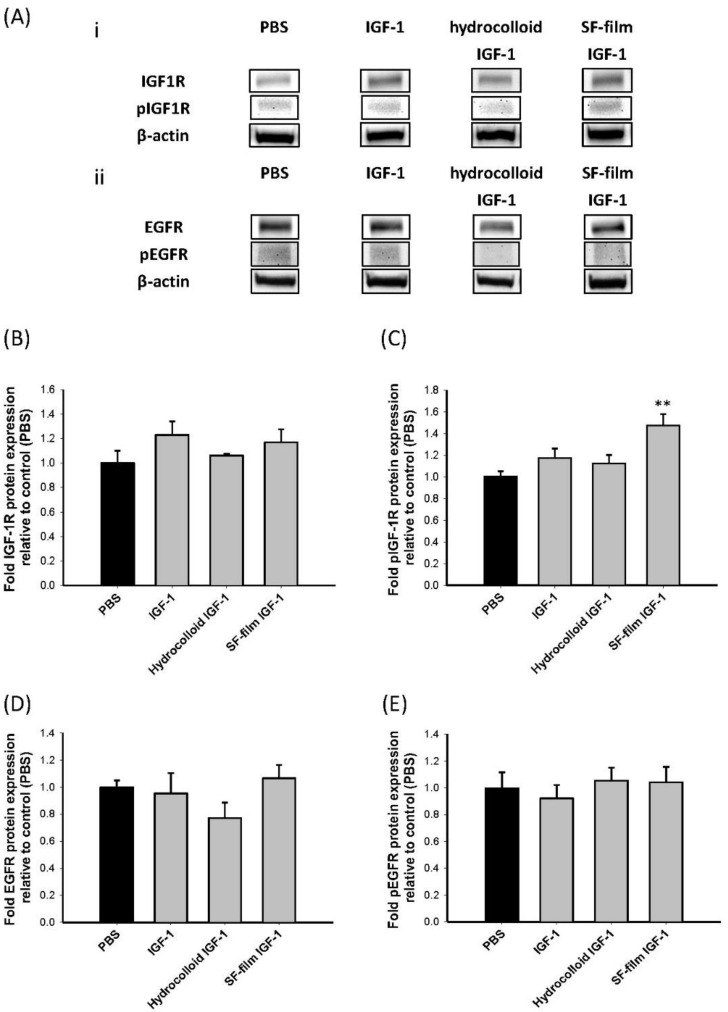
Immunoblotting for insulin-like growth factor type 1 receptor (IGF1R) or epidermal growth factor receptor (EGFR) and phosphorylated IGF1R or EGFR pathway from wound tissues of diabetic mice following treatment with PBS (control), native insulin-like growth factor 1 (IGF-1), a commercial dressing product (hydrocolloid IGF-1), and IGF-1-loaded SF film (SF film IGF-1) treatments. (**A**) Representative Western blot of *db*/*db* mice treated with different treatments 13 days post-wounding. (i) represents IGF-1 receptor (IGF1R) and phosphorylated IGF1R protein expression. (ii) represents EGF receptor (EGFR) and phosphorylated EGFR protein expression. Actin was used as a loading control. Quantification of (**B**) IGF1R protein expression; (**C**) phosphorylation of IGF1R; (**D**) EGFR protein expression; and (**E**) phosphorylation of IGF1R. Significant differences between control (PBS) and other treatments were determined using Dunnett’s multiple comparison post hoc test. ** *p* < 0.01; *n* = 5; ±SEM.

We verified whether the IGF-1 released from SF films contributed to the stimulatory effects shown on wound healing in *db*/*db* mice by analyzing the expression and phosphorylation levels of IGF1R and epidermal growth factor receptor (EGFR), which are involved in the wound epithelialization-related signaling pathways (Figure 7A). Expression levels of IGF1R were approximately the same in all treatments (Figure 7B), while a significant enhancement (1.47 times) in the IGF1R phosphorylation level was observed following SF film IGF-1 treatment when compared to the PBS control, native IGF-1, and IGF-1-loaded hydrocolloid treatments (Figure 7C). In addition, no significant changes in EGFR expression and phosphorylation were noted for any of the treatment groups (Figure 7D,E). These results indicated that the promotion of wound recovery in *db*/*db* mice was related to the enhancement of the IGF1R phosphorylation level in SF film IGF-1 treatment.

## 3. Discussion

### 3.1. Drug Delivery for Wound Healing

Wound healing is a dynamic physiological process consisting of several phases, including: (i) coagulation and hemostasis, (ii) inflammation, (iii) proliferation, and (iv) remodeling [34]. Growth factors play a major role in wound healing as they stimulate the proliferation and migration of several cell types, as well as the production of the extracellular matrix [35,36]. Nevertheless, certain conditions, such as diabetes, characterized by poor microcirculation prohibit the effective delivery of growth factors for chronic wound repair [37]. In addition, many growth factors exhibit a short half-life in vivo. Therefore, it is generally believed that a constant supply of growth factors to the localized wound bed via slow-release drug delivery would be beneficial in chronic wound therapy [38,39]. The findings observed in this study are also strongly in support of this theory, as evidenced by the greater diabetic wound healing due to the sustained release of the IGF-1 growth factor in the IGF-I loaded SF films treatment group.

### 3.2. SF as a Drug Delivery Material

In the last two decades, SF has been proved to be an excellent material carrier for the delivery of a variety of bioactive compounds including amino acids and growth factors [40,41,42]. For instance, kindling epileptogenesis was controlled for one week using an SF-based release system combined with adenosine augmentation therapies for refractory epilepsy [41]. One important and potentially useful property of SF as a delivery matrix relies on its capability in prolonging the half-life of the growth factor complexed with it. The activity of human acidic fibroblast growth factor could be extended for more than 15 days when delivered via a heparinized SF hydrogel, which can also facilitate the migration of fibroblast cells and the healing of full-thickness skin excision in rats [42]. Similarly, the half-life of nerve growth factor embedded with SF-based nerve conduits could be prolonged for at least 3 weeks [43]. Insulin also exhibits approximately a twice longer half-life in SF–insulin bioconjugate form than in its native soluble form [44]. However, no significant difference was noted in wound closure among SF film, SF film loaded with EGF/silver sulfadiazine, and hydrocolloid [45]. The current study demonstrates that the SF films fabricated using the presented method exhibit a sustained release ability of IGF-1, preserving its activity over than 30 days. Such properties will surely benefit chronic wound healing, which commonly takes several months for complete recovery.

The growth factor release kinetics of SF films may depend on the composition of SF structure, mixture, or drug type. It has been reported previously that SF films subjected to air-drying and water vapor annealing transform their amorphous structure into a high crystallinity beta-sheet content [46,47,48]. The increase in crystallinity in the silk polymer matrix has been shown to be advantageous for specific drug loading [23,49]. According to previous reports, it has been hypothesized that the binding of silk fibroin protein and substrate is attributed to hydrophobic interactions as well as partial electrostatic interactions [50,51]. For long-term IGF-1 release, we established a novel patented procedure to improve the insolubility and biocompatibility of SF film by modulating its crystalline structure. Besides several superior physical properties such as transparency and mechanical strength, the SF films obtained by our method exhibit an excellent property in IGF-1 delivery—prolonging the IGF-1 half-life for more than 2000 times and showing a sustained release for more than 30 days. More importantly, the IGF-1 protection and slow-release activities are also reflected in the promotion of the wound healing in vivo.

### 3.3. Release Kinetics of IGF-1 from SF

In pharmacokinetic studies, the drug release mechanism can typically be evaluated based on the exponent *n*. If *n* = 0.5, the release mechanism is considered Fickian diffusion; if *n* = 1, the release mechanism belongs to Case-II release, with the drug release rate corresponding to zero-order release kinetics [52]. For anomalous release behaviors between Fickian and Case-II release, the *n* value is generally between 0.5 and 1 [28]. A zero-order release profile is typically preferred in drug delivery because the drug could be maintained at a constant concentration and hence the frequency of drug administration and side effects could be reduced [53]. It has been demonstrated that the drug release kinetics varies widely and depends on factors such as the molecular weight, composition, hydrophobicity, and ionization of the drug and its carrier [52]. As far as we are aware, there is no clinically established range of IGF-1 for wound therapy. Therefore, we performed a pilot test first (data not shown) and the results showed that the optimal range was between 6.5 pmol and 65 pmol. In this study, we further achieved a zero-order release kinetics of IGF-1 using SF film for at least 19 days even when relatively low amounts of IGF-1 (0.65 and 6.5 pmol) were used. Similar zero-order kinetics for SF-based delivery results was also noted in previous studies [41,54]. The delivery of small molecular drugs such as adenosine has been tested on the SF film using drying and crystallization processes. The release mechanism of adenosine loaded into SF was close to zero-order kinetics for 14 days [55].

### 3.4. Drug Release Model of SF

When relatively low amounts of IGF-1 (0.65 and 6.5 pmol) were used to load into SF films, approximately 90% and 75%, respectively, of IGF-1 were released exponentially from SF films till 19 days at 37 °C before achieving a slow and stable diffusion rate. SF films loaded with fluorescein-iso-thio-cyanate (FITC)-dextran with a 4 to 40 kDa molecular weight also fitted the two-phase release profile, the exponential and diffusion-based model, in an earlier report [50]. On the other hand, a higher amount of IGF-1 (65 pmol) loaded into the SF film sustained the exponential release till the end of the study. For a scaffold of low porosity such as the SF film manufactured in our study, it has been proposed that the drug release commonly and primarily is based on the heterogeneous hydrolysis of the surface domain, and other factors such as diffusion, polymer swelling and polymer degradation play a secondary role [21,56]. Many factors during fibroin degumming, purification, and film-forming may alter the characteristics of SF films, thereby affecting the drug loading and drug release model predicted for SF films [57,58,59,60]. Thus, in addition to loading optimal amounts of the drug payload, sustained exponential release of the drug may be prolonged through an improved manufacturing procedure to control the agent’s dosage.

### 3.5. In Vitro and In Vivo Diabetic Wound Healing Studies

To evaluate the IGF-1 loaded with SF film in diabetic wound healing, we determined the effect of IGF-1 alone, hydrocolloid IGF-1, and SF film IGF-1 in BALB/3T3 cells firstly. Although, compared to the control treatment, the rate of cell scratch closure was enhanced in IGF-1 and SF film IGF-1 treatments, the effect of wound healing reflected no significant difference between IGF-1 and SF film IGF-1 in vitro short-term cell life. However, it is somewhat satisfying to find that the long-term wound healing in diabetic mice, as well as other internal wound parameters, including blood vessels, wound edge, granulation tissue, and re-epithelialization, were significantly improved in the experimental group treated with SF film loaded with IGF-1 compared to free IGF-1 or hydrocolloid loaded with IGF-1. The finding is in good agreement with several earlier studies that also implicated the promoting activity of SF in re-epithelialization, tissue formation, and regeneration of corneal epidermal and chronic wound healing [6,61,62,63]. All these results strongly suggest that SF film is a useful biomaterial in accelerating diabetic wound healing.

Moreover, IGF-1 is a well-known stimulator of keratinocyte growth and migration, key properties for wound re-epithelialization [18,64]. In addition, IGF-1 facilitated the activation of the PI3-kinase (PI3K)/Akt, a key player in the signaling of angiogenesis [19]. In the current study, we also observed an increase in blood vessels in granulation tissue following treatment with the IGF-1-loaded SF film. Angiogenesis was less evident in wounds treated with free IGF-1, presumably because the growth factor was rapidly degraded in vivo [65], thereby limiting its effect on wound repair.

### 3.6. Molecular Mechanisms of Wound Healing by IGF-1-Loaded SF Films

**Figure 8:**
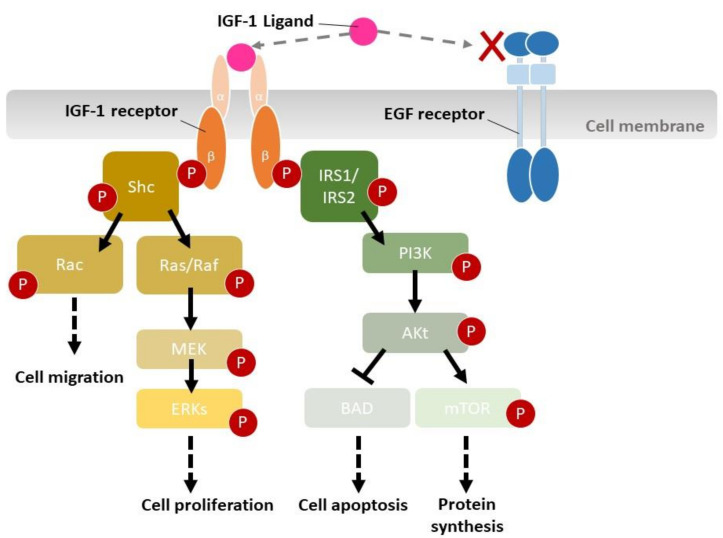
Schematic representation of insulin-like growth factor 1 (IGF-1) triggering the insulin-like growth factor type 1 receptor (IGF1R)-related signal transduction pathway. Activation of the IGF-1 receptor (IGF1R) triggers several signaling pathways. IGF1R, a tyrosine kinase, auto-phosphorylates multiple cellular proteins, including members of the Shc, Rac, Ras/Raf, and IRS1/IRS2 families. Considering phosphorylation, Shc was activated to regulate Rac and Ras/Raf. The Rac pathway is important for cell migration, while the Ras/Raf pathway is critical for proliferative responses. IRSs interact with signaling molecules, while activation of the PI3K and Akt pathways prevents apoptosis, also triggering protein synthesis.

The current study also analyzed the expression levels of IGF1R and EGFR, two major players involved in the regulation of epidermal cell growth and migration [66]. Immunohistochemistry analysis of the wound sections revealed that SF film loaded with IGF-1 only downregulated IGF1R phosphorylation levels, while EGFR phosphorylation and IGF1R and EGFR expression levels were similar among all treatments. A number of previous studies have also demonstrated that in diabetic mice, both IGF1R and EGFR could be activated even when insulin signaling was interrupted, and the activation of IGF1 and EGF pathways led to the enhancement of keratinocyte migration [18,67,68]. Our study provides strong evidence showing that IGF1R phosphorylation is enhanced by IGF-1, which is released continuously from the SF film. This was followed by the down-regulation of IGF1R-related signal transduction pathways [69], such as Shc, IRSs, PI3K, and Akt, eventually leading to the acceleration of cell migration, proliferation, protein synthesis, and wound repair in *db*/*db* mice (Figure 8).

### 3.7. Wound Healing Effectiveness between Hydrocolloid and SF Films

Hydrocolloid is a common dressing material which forms a moist environment for wound repair. Although hydrocolloid is a useful dressing in wound healing, it absorbs IGF-1 strongly, resulting in very little IGF-1 release and less significant wound healing than using IGF-1-loaded SF films. A similar growth factor release profile has been reported previously for hydrocolloid [70], and therefore, it is not surprising to find that the hydrocolloid dressing alone lacks a healing function in diabetic wounds both in experimental animals [71,72] and in diabetes patients [73,74]. The continued presence of critical IGF-1 concentrations in the wound bed has been shown to stimulate wound repair significantly in diabetic animal models [17,75]. A previous study also indicated that human mesenchymal stem cell differentiation and tissue regeneration were enhanced through IGF-1 released from an SF scaffold accompanied with the acceleration of wound repair [29]. Although IGF-1 is thought to enhance cell proliferation primarily, the wounds of *db*/*db* mice were improved during all wound phases using our dressing. Several earlier reports also showed that silk fibroin could optimize platelet adhesion, aggregation, anti-inflammation, and scar prevention [76,77,78]. Therefore, we propose that the IGF-1-loaded SF film is useful for all wound healing phases.

## 4. Materials and Methods

### 4.1. Preparation of an SF Aqueous Solution

Silkworms (*Bombyx mori* L.) were reared in a temperature-controlled environment (26–28 °C) in our institution (Taiwan Silkworm Germplasm, Miaoli District Agricultural Research and Extension Station, Miaoli, Taiwan) for cocoon production. The SF degumming protocol was modified from previously described procedures [79,80]. Briefly, cocoons were boiled in deionized water for 3 h to remove sericin from raw silk. Next, degummed silk was dried at 75 °C, and then dissolved in 9.3 mol/L lithium bromide (FERAK, Berlin, Germany) at 60 °C for 6 h. The aqueous fibroin solution was dialyzed against deionized water using a dialysis membrane (MWCO 6000–8000 Da, Spectra/Por, New Brunswick, NJ, USA) for 48 h at room temperature.

### 4.2. Preparation of IGF-1-Loaded SF films

Fabrication of water-insoluble SF films was performed as previously described [46] with minor modifications, and the novel patented procedure is illustrated in Figure 1. The SF solution was cast onto a dish previously coated with polydimethylsiloxane (PDMS) (Bio-cando, Taoyuan, Taiwan) and allowed to air dry. The film was then water-annealed in a water vapor environment to enhance beta-sheet crystallinity. Water-insoluble SF films and commercial 3M ^TM^ Tegaderm ^TM^ hydrocolloid wound dressings (hydrocolloid) (3M, Maplewood, MN, USA) were cut into 10-mm diameter circles and then sterilized using UV irradiation. The dried SF film was approximately 50–70 μm thick.

### 4.3. Release Kinetics of IGF-1-Loaded SF Films

IGF-1 used in this study was purchased from Sigma-Aldrich, St. Louis, MO, USA (Cat. #SRP3069). The release kinetics of IGF-1-loaded SF films were determined using a previously published procedure [81]. We chose 37 °C to simulate the condition of human wounds and 4 °C to determine whether the temperature is suitable for storage of IGF-1-loaded SF film for clinical uses. First, the dressings were incubated in 5 mL of IGF-1 of different concentrations (0.65 pmol, 6.5 pmol, and 65 pmol) with gentle shaking at 4 °C and 37 °C for 816 h (34 days). Binding of IGF-1 to SF films or hydrocolloid was determined by measuring IGF-1 depletion from the solution using an IGF-1 DuoSet ELISA kit (R&D Systems, Minneapolis, MN, USA). The IGF-1 loaded SF films and hydrocolloid dressing were transferred to 5 mL of phosphate-buffered saline (PBS) and the cumulative IGF-1 release was then determined at different time points using ELISA.

The Ritger-Peppas equation, which is used to describe Fickian and non-Fickian release behavior [52], was applied to the kinetic study to investigate the IGF-1 release mechanism from SF films in this study. The equation 1 is as follows:

(1) MtM∞=Kt n

where *M_t_* is the amount of IGF-1 released at time *t*; *M∞* is the total amount of IGF-1 released at infinite time; *K* is the release constant; *n* is the release exponent.

### 4.4. In Vitro and In Vivo Wound-Healing Studies

Mouse BALB/3T3 fibroblasts were cultured in DMEM with 50 mM D-glucose and 10% FBS (5% CO_2_/37 °C). The cells scratch closure was investigated by CytoSelect ™ Wound Healing Insert Kit (Cell Biolabs, San Diego, CA, USA) according to the manufacturer’s protocol. Wound closure was imaged post scratching using an inverted fluorescence microscope (ECLIPSE Ts2-FL, Nikon, Tokyo, Japan). Cell cytotoxicity was calculated by LIVE/DEAD™ Viability/Cytotoxicity Kit (Thermo Fisher Scientific, Waltham, MA, USA) and examined under a fluorescence microscope. The cell viability was measured with alamarBlue reagent (Thermo Fisher Scientific, Waltham, MA, USA) according to the formula provided by manufacturer.

Murine wound healing model assays were approved by the Institutional Animal Care and Use Committee of the National Taiwan University (IACUC Approval No: NTU108-EL-00071). Six-week-old BKS.Cg-*Dock7 ^m^ +*/*+ Lepr ^db^*/JNarI (*db*/*db*) female mice were obtained from the National Laboratory Animal Center of Taiwan (Taipei, Taiwan). Mice were housed in polycarbonate animal cages with hardwood bedding at 21 ± 1 °C and 12 h/12 h light/dark cycles and had free access to water and food. Prior to the experiment, the plasma glucose level of each mouse was tested; those with a blood glucose level above 300 mg/dL were chosen for further analysis. Mice were anesthetized using isoflurane and 8 mm-diameter, full-thickness wounds were generated. Mice were separated into four treatment groups; each wound received one of the following treatments: (A) 5 μL of PBS (control group); (B) 5 μL PBS containing 65 pmol IGF-1; (C) a circular 10-mm-diameter hydrocolloid dressing loaded with 65 pmol IGF-1 (hydrocolloid IGF-1); and (D) a circular 10-mm-diameter SF film containing 65 pmol IGF-1 (SF film IGF-1). Treatments were applied directly to the wound bed and as a fixed layer were covered with a sterile 3M ^TM^ Tegaderm ^TM^ transparent film (3M, Maplewood, MN, USA). Wound condition was measured and recorded every 2–3 days. Mice were sacrificed and wound tissues were harvested for further histology and protein analysis 13 days post-wounding.

### 4.5. Histological Analysis of Wound-Healing

For histological analysis, tissues were fixed in formalin, embedded with paraffin, sliced into 5 μm sections, and stained using hematoxylin (3008-1, Muto Pure Chemicals, Tokyo, Japan) and eosin (32042, Muto Pure Chemicals, Tokyo, Japan) (H&E). Some tissue sections were deparaffinized and rehydrated prior to staining with CD31 antibody (GTX130274, Genetex, Hsinchu, Taiwan) using an automated immunohistochemistry system (BOND-MAX, Leica, Wetzlar, Germany). These were then visualized under a light microscope (IM-3, OPTIKA, Ponteranica, Italy). Wound parameters including wound edge distance, epithelial gap, granulation tissue area, re-epithelialization, epithelial tissue area, and adipose tissue gap were calculated from five individual mice.

### 4.6. Immunoblotting Assay

Total proteins were extracted from the skin wound tissue of *db*/*db* mice using RIPA lysis buffer (Cell Signaling, Danvers, MA, USA) containing an ethylenediaminetetraacetic acid-free protease inhibitor cocktail (Roche Diagnostics, Rotkreuz, Switzerland). Protein extracts were resolved on a 4–20% gradient sodium dodecyl sulfate (SDS)–polyacrylamide gel (NuSep, Germantown, MD, USA) and transferred onto a nylon membrane. Membranes were blocked for 1 h with 5% BSA in Tris buffer-saline (pH 7.6) containing 0.1% Tween-20 (TBST), and then incubated with the following primary antibodies: anti-insulin-like growth factor-1 receptor (IGF1R) antibody (#9750, Cell Signaling, Danvers, MA, USA), anti-phosphorylation of IGF-1 receptor (pIGF1R) antibody (Cell Signaling, Danvers, MA, USA), anti-epidermal growth factor receptor (EGFR) antibody (Genetex, Irvine, CA, USA), anti-phosphorylation of epidermal growth factor receptor (pEGFR) antibody (Cell Signaling, Danvers, MA, USA), and anti-actin antibody (Santa Cruz, Dallas, TX, USA) for 2 h at 20 ± 1 °C. After washing with TBST, membranes were incubated with a horseradish peroxidase (HRP)-conjugated secondary antibody (Santa Cruz, Dallas, TX, USA) for 2 h at 20 ± 1 °C followed by membrane incubation in an enhanced chemiluminescent (ECL) detection system (WBKLS0500, Merck Millipore, Burlington, MA, USA) for target antigen visualization. Intensity of targeted protein bands was quantified and normalized to the actin expression area using ImageJ 1.8.0v software (National Institute of Health, Bethesda, MD, USA) 

### 4.7. Statistical Analyses

Kinetic, in vitro, and in vivo studies were calculated with a minimum of *n* = 5 for each treatment. Statistical analyses were performed using SAS-EG 7.1 software (SAS Enterprise Guide, SAS Institute Inc., Cary, NC, USA). One-way ANOVA, followed by Dunnett’s multiple comparison post hoc test, was used to compare statistically significant differences (* *p* < 0.05, ** *p* < 0.01, and *** *p* < 0.001) among control and experimental samples. All error bars represent the standard error of the mean (SEM).

## 5. Conclusions

In the current study, we have established a method to produce highly pure SF films, which served as an ideal matrix for IGF-1 sustained release following zero-order pharmacokinetics. We have also confirmed that the cell growth promoting activity of IGF-1 was preserved after being complexed with the SF films, thereby significantly prolonging the half-life of IGF-1. We further demonstrated that the effectiveness of IGF-1-loaded SF films in vitro and in vivo diabetic wound healing was likely related to the sustained release of IGF-1 from the SF films. Finally, evidence was provided that the released IGF-1 triggered the down-regulation of IGF-1 receptor phosphorylation, thereby accelerating the downstream wound repair process such as cell migration. Together, we believe that SF films fabricated using the protocol presented in the current study are an excellent vehicle for sustained drug delivery that will find important applications in wound healing as well as other applications such as tissue regeneration and organ reconstruction.

## 6. Patents

Lin, M.-J. and Lu, M.-C. are coinventors of a patent (Taiwan patent pending number: 109138288) application pertaining to the SF film system disclosed in the manuscript.

## Figures and Tables

**Table 1:** Parameters obtained from Ritger and Peppas equation for IGF-1 release from SF films.

Loading Amount of IGF-1 (pmol)	Temperature (°C)	K ^b^ (% h^−1^)	*n* ^c^	R^2 d^	Time ^e^ (h)
0.65	4	0.03	1.03	0.92	48–816
6.5		0.05	0.97	0.96	48–816
65		0.06	1.02	0.88	48–816
0.65	37	0.11	1.21	0.95	48–456
6.5		0.23	1.00	0.96	48–456
65		0.09	1.05	0.98	48–816

^a^ Ritger-Peppas equation was defined as follows: Mt/M∞ = Kt *^n^*; where Mt is the amount of IGF-1 released at time t; M∞ is the total amount of IGF-1 released at infinite time; *n* is the release exponent. ^b,c^ K and *n* values are the release and constant exponent, respectively, which are calculated using the Ritger-Peppas equation. ^d^ R^2^ values are linear regression coefficient values. ^e^ Time means the vector of the corresponding time value.

## Data Availability

The datasets corresponding to the current study are available from the corresponding author upon request.

## Supplementary Materials

The following are available online at https://www.mdpi.com/article/10.3390/ijms22126267/s1.
