# Supplementary material for: Sustained Release of Insulin-Like Growth Factor-1 from Bombyx mori L. Silk Fibroin Delivery for Diabetic Wound Therapy
Source: Int J Mol Sci. 2021 Jun 10;22(12):6267. doi: 10.3390/ijms22126267 (PMC8230471; doi:10.3390/ijms22126267)
Supplement: Supplementary file 1 [file ijms-22-06267-s001.zip › ijms-1235218-supplementary.pdf]

International Journal of
Molecular Sciences

MDPI

Supplementary Materials

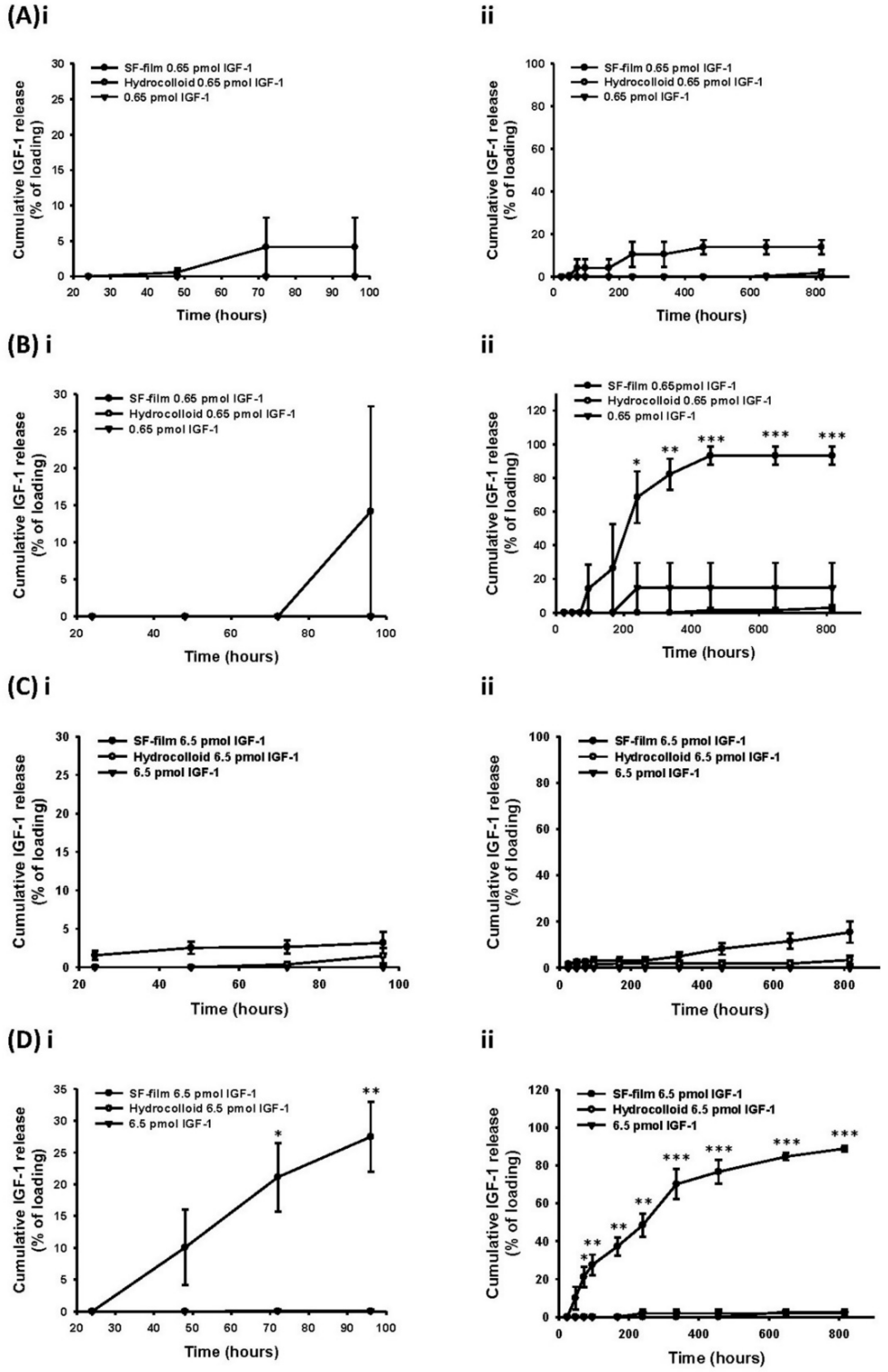

Figure S1. Release profiles of insulin-like growth factor 1 (IGF-1)-loaded silk fibroin-film (IGF-1-loaded SF-film) IGF-1 loaded onto SF-films or hydrocolloid dressing. (**A**) The cumulative release curve of 0.65 pmol IGF-1 (i) from 24 to 96 h, and (ii) 24 to 816 h at 4 °C. (**B**) The cumulative release curve of 0.65 pmol IGF-1 (i) from 24 to 96 h, and (ii) 24 to 816 h at 37 °C. (**C**) The cumulative release curve of 6.5 pmol (i) from 24 to 96 h, and (ii) 24 to 816 h at 4 °C. (**D**) The cumulative release curve of 6.5 pmol IGF-1 (i) from 24 to 96 h, and (ii) 24 to 816 h at 37 °C. (●) SF-films loaded with IGF-1; (○) Hydrocolloid loaded with IGF-1; (▼) IGF-1 alone, *$p < 0.05$, **$p < 0.01$, ***$p < 0.001$ in comparison with IGF-1 alone treatments ($n = 3$, ± SEM) using a Dunnett's t test.
